# Prospective comparison of the digestive tract resistome and microbiota in cattle raised in grass-fed versus grain-fed production systems

**DOI:** 10.1128/msphere.00738-24

**Published:** 2025-02-14

**Authors:** Jiye Kwon, Windy Tanner, Yong Kong, Martina Wade, Chad Bitler, Marilia B. Chiavegato, Melinda M. Pettigrew

**Affiliations:** 1 Department of Epidemiology of Microbial Diseases, Yale School of Public Health, New Haven, Connecticut, USA; 2 Public Health Modeling Unit, Yale School of Public Health, New Haven, Connecticut, USA; 3 Department of Biostatistics, Yale School of Public Health, New Haven, Connecticut, USA; 4 Bioinformatics Resource at the W.M. Keck Foundation Biotechnology Resource Laboratory, Yale School of Medicine, New Haven, Connecticut, USA; 5 Greenacres Foundation Inc., Cincinnati, Ohio, USA; 6 Departments of Horticulture and Crop Science and Animal Sciences, The Ohio State University, Columbus, Ohio, USA; 7 Department of Environmental Health Sciences, University of Minnesota School of Public Health, Minneapolis, Minnesota, USA; Nanjing University of Chinese Medicine, Nanjing, Jiangsu, China

**Keywords:** microbiota, resistome, antibiotic resistance, bovine

## Abstract

Most antimicrobials sold in the United States are used in food animals. Farm management practices contribute to antibacterial resistance (AR). Controversially, grass-fed diets have been recommended over grain-fed diets to reduce AR in beef cattle. Ionophore feed additives (non-therapeutic antibiotics that enhance feed efficiency) may contribute to AR development. We used shotgun metagenomic sequencing of fecal swabs to prospectively compare the cattle gastrointestinal resistome and microbiota in two different production systems over five periods from pre-weaning to pre-harvest. Cattle were grass-fed and pasture-raised (system A, *n* = 33) or grain-fed with ionophore additives in feedlots (system B, *n* = 34). System A cattle averaged 639 lb and 22.8 months of age, and system B cattle averaged 1,173 lb and 12.4 months of age preharvest. In total, 367 antibiotic resistance genes (ARGs) and 329 bacterial species were identified. The resistome of system A cattle had higher alpha diversity than system B cattle over their lifespan (*P* = 0.008). Beta-diversity estimates indicated overlap in the pre-weaning resistome and microbiota in both systems, which diverged post-weaning, with increases in several medically important ARGs when system B cattle transitioned to a grain diet. Analysis of compositions of microbiomes with bias correction indicated that levels of tetracycline, macrolide, aminoglycoside, beta-lactam, and bacitracin ARGs were significantly higher in system B cattle pre-harvest. Resistome changes were highly correlated with bacterial community changes (Procrustes, *M*^2^ = 0.958; *P* = 0.001). Potentially modifiable farm management strategies, including diet and ionophores, may influence abundance and diversity of ARGs in fecal samples from cattle.

**IMPORTANCE:**

Antibiotic resistance is a One Health threat. More antibiotics are used in agriculture than in human medicine. We compared the relative abundance of antibiotic resistance genes (ARGs) and bacterial species in cattle raised in two different cattle production systems (grass- and grain-fed). Fecal swab samples were collected at five time points spanning pre-weaning and prior to harvest. The antibiotic resistance gene and bacterial communities were relatively similar in the pre-weaning period when cattle in both systems were milking and on pasture. Resistance genes and bacterial communities diverged post-weaning when system B cattle were given a grain diet with feed additives for growth promotion containing non-medically important antibiotics (i.e., ionophores). The levels of medically important ARGs (e.g., macrolides) increased in system B grain-fed cattle post-weaning and were higher than in system A just prior to slaughter. These data provide additional evidence that farm management strategies impact the level of antibiotic resistance.

## INTRODUCTION

Antibiotic resistance is a major global One Health threat (1, 2). The excessive use and misuse of antibiotics are considered a major, but not the sole, driver of resistance (3). Over 70% of antimicrobials sold worldwide are used in livestock to enhance growth, health, and productivity (4, 5). The use of antibiotics in livestock has been linked to drug-resistant infections in animals (6). Resistant bacteria can also spread between animals and people (7); however, some studies suggest limited transmission of bacteria and resistance determinants between livestock and humans (8, 9).

Several international organizations, including the World Health Organization and the Food and Agriculture Organization of the United Nations, have issued statements calling for worldwide efforts to decrease antimicrobial use across One Health sectors (10). The US Food and Drug Administration (FDA) Center for Veterinary Medicine issued directives in 2017 that require veterinary oversight for antibiotic use and restrict their use to purposes that ensure animal health; the directives also put an end to the use of medically important antibiotics for growth promotion and feed efficiency (11).

Grass-fed cattle and other regenerative farming methods have been proposed as a way to reduce antibiotic use and improve One Health in comparison to conventional farming methods (12). Grass-fed cattle that are raised on pasture generally require fewer antibiotics than conventionally raised grain-fed cattle (12). Conventionally raised cattle are often finished in feedlots in close proximity to each other, which could affect transmission of antibiotic-resistant bacteria between animals (13, 14). Moreover, ionophores, which are antibiotics that are not considered medically important for human health, are widely used as a feed additive to increase growth and feed efficiency in conventional grain-fed cattle production systems (15, 16). Understanding of ionophore resistance is incomplete, and One Health consequences of their use are unclear (16).

A limited number of studies have attempted to characterize the impact of farm management practices, including antibiotic use, on the beef cattle gut resistome (17, 18). Noyes et al. prospectively examined the gastrointestinal resistome of commercial beef cattle from feedlot entry to slaughter and showed that the diversity of ARGs decreased over time (17). The ARGs that were lost were mainly those that encoded resistance to antibiotics that were not used in the production system during the study period; thus, these data suggest that antibiotic selection pressure plays a key role in the maintenance of ARGs in beef cattle (17, 18). Vikram et al. conducted a comprehensive assessment of ARGs in commercial beef cattle raised with and without antibiotics (18). While the abundance of beta-lactam, macrolide–lincosamide–streptogramin B (MLS), aminoglycoside, and tetracycline ARGs was higher in cattle raised with antibiotics, shotgun metagenomic analyses indicated that the total aggregated abundances of ARGs did not significantly differ between cattle raised in the two systems (i.e., with and without antibiotics) (18). These studies highlight the complexities of antibiotic resistance, and there is no clear consensus on the optimal farm management strategies to reduce the prevalence of antibiotic resistance (18, 19). Moreover, few if any studies have compared grass- and grain-fed cattle production systems and their impact on the cattle gastrointestinal resistome and microbiota.

The goal of this study was to compare the gastrointestinal resistome and microbiota in cattle raised in two different production systems. System A was a regenerative farm that raised grass-fed cattle on pasture, and system B was an agricultural research station that served as a model for conventional farming. System B cattle were grain-fed, provided with ionophores to improve feed efficiency, and finished in feedlots. Fecal swabs were prospectively collected from cattle in both systems at five time points, and shotgun metagenomic sequencing data were used to compare the gastrointestinal resistome and microbiota in cattle in the two systems. These data provide insight into the impact of different farm management practices, regenerative (i.e., grass-fed) and conventional (i.e., grain-fed), on the burden of antimicrobial resistance.

## MATERIALS AND METHODS

### Study location and design

The two production systems were located in Ohio, USA. System A practiced regenerative agriculture; cattle were raised on pasture and were 100% grass-fed and finished. Fescue and a range of cool and warm season grasses, forbs, and legumes formed the main forage base in system A. Hay was fed in winter and predominantly fescue, orchard grass, timothy grass, and clovers. The cattle were rotated through approximately 30 pastures on two farms; the central latitude and longitude coordinates were 39°13′23.36″N, 84°20′33.62″W, and 38°50′37.43″N, 84°1′33.64″W. We identified 33 registered Black Angus calves, 10 heifers, and 23 steers in system A. Calves were alongside their mothers on pasture and weaned anywhere from 6 to 12 months of age, with the average weaning occurring at approximately 210 days (about 7 months) of age. System A cattle remained on pasture until harvest. System A cattle received two vaccines: Cattlemaster FP5L5, which provides protection against four respiratory viruses, diarrhea, and leptospirosis, and Covexin 8, which provides protection against *Clostridium* species. System A cattle did not receive any systemic antibiotics for illness during the study. There was some attrition over time; one system A steer died, and one steer and one heifer were sold prior to harvest.

The second study site, system B, served as a model for conventional/intensive farming. The latitude and longitude coordinates for system B were 39°47′13.89″N and 81°31′11.22″W. Thirty-four spring-born Angus × SimAngus-crossbred calves, all steers, were identified in system B. Pre-weaning calves were alongside their mothers and given free access to grass in pasture. Calves were weaned at 125 days (about 4 months), on average, placed in feedlots and transitioned to a grain diet that included feed additives containing the ionophore Rumensin, also known as monensin. The cattle were randomized to high and low grain diet groups. PERMANOVA analysis of ARG data showed no significant differences in ARG composition across these groups (*P* = 0.994; 10,000 permutations). Thus, we analyzed the cattle in system B as a single “grain-fed” group. Additional details of the grain diet are in Tables S1 to S3. System B cattle received the following vaccines: Ultrachoice 7, which provides protection against *Clostridium* species, BoviShield GOLD FP5 L5, which provides protection against four respiratory viruses, diarrhea, and leptospirosis, and an autogenous vaccine for pinkeye.

Fecal swab samples were obtained from cattle in both systems at a minimum of five time points (Fig. S1). Samples were individually collected from system A cattle pre- and post- weaning and at additional time points selected to capture seasonal changes in the type and quality of grass available for their diet. The fecal swab sampling scheme and names for system A cattle were as follows: pre-weaning (S1), post-weaning (S2), summer (S3), winter (S4), and pre-harvest (S5). Pre-harvest samples were collected the day prior to harvest when the cattle reached the appropriate weight for slaughter. The sampling scheme for system B involved collection of fecal swabs at four dietary stages: pre-weaning (S1), transition (S2), backgrounding (S3), and finishing (S4) diets; pre-harvest fecal swabs (S5) were also collected. FecalSwabs with Cary-Blair transport medium (Copan, Brescia, Italy) were used and the samples were collected by inserting a swab approximately 2 to 5 cm into the anus of each animal. Swabs were placed in transport media and stored at 4°C and shipped overnight to the Yale School of Public Health where they were stored at −80°C until DNA extraction.

### Data and sequencing

DNA extraction and shotgun metagenomic sequencing was done as previously described (20). DNA was extracted from 329 fecal swabs using the PureLink Microbiome DNA Purification Kit (Invitrogen Carlsbad, CA). Shotgun sequencing libraries were prepared as per the manufacturer’s instructions using the NEBNext Ultra II FS DNA Library Prep Kit with Sample Purification Beads for Illumina. Individual samples were barcoded for identification and the libraries pooled in approximately equal nanogram amounts. Samples were sequenced in batches using a 150 bp paired-end sequencing protocol at the Yale Center of Genome Analysis on the Illumina NovaSeq.

### Bioinformatic and statistical analyses

Shotgun metagenomic sequence reads were sorted, trimmed, and low-quality reads were filtered using Btrim software version 0.3.0 (21). Of the 329 sequenced samples, there were two that could not be distinguished from each other due to a laboratory barcoding or sequencing error. These were samples S2 and S3 from one animal (ID H505) in system A. The reads from these two individual samples were removed from subsequent bioinformatic and statistical analyses. The average number of total paired reads per fecal sample and standard deviation (SD) for the remaining 327 samples was 39,454,075 ± 13,152,689. After trimming, the mean number and SD of reads were 37,705,707 ± 12,592,550. The total number of paired reads, trimmed pairs, and classified reads for each sample are provided in Table S4. We used the ARG online analysis pipeline with the expanded structured ARG (SARG) database and software v2.0 for classification, quantification, and normalization of ARGs (22). The ARGs were classified at the type level (antibiotic class) and subtype level (individual gene) (22). ARG abundances were normalized to prokaryotic cell numbers, which were determined by calculating the average coverage of a set of universal single-copy bacterial genes (22, 23). The ARG abundances were provided in units of resistance genes per prokaryotic cell. We used the MetaPhlAn3 pipeline for taxonomic identiﬁcation of shotgun metagenomic sequence reads and to estimate the relative abundance of taxa within each sample (24, 25).

We used R 4.3.3 (R Foundation for Statistical Computing) for statistical analyses and ggplot2 for data visualization (26). We uniformly removed all-zero features and applied 10% minimum prevalence filtering for ARG subtype level and taxonomic analyses. For heatmap visualization of ARG subtypes, an additional filter was applied using a minimum abundance of 0.05. All taxa with a variance less than 0.001 were removed before taxa heatmap visualization.

Beta-diversity was calculated using Bray–Curtis dissimilarity indices, which are used to measure the compositional dissimilarity between samples (27, 28). Bray–Curtis dissimilarity indices were used for the creation of ordination plots, and nonmetric multidimensional scaling (NMDS) ordination was used to graphically depict differences in ARG community and taxonomic community profiles (29). We used two different measures of alpha diversity (30). The Shannon diversity index provides a measure of the number of different types of ARGs (or taxa) within each sample and takes their abundance and relative distribution into account (30, 31). The inverse Simpson diversity index is a probability that gives more weight to abundant ARGs (or taxa) (30, 31).

Unadjusted associations between relevant characteristics were compared by *χ²*, Wilcoxon rank-sum, and Wilcoxon signed-rank tests as appropriate. We used Cohen’s *d* as a measure of the effect size to compare alpha diversities in pre-weaning and pre-harvest samples for each system separately (32). We employed an inverse Gaussian generalized linear mixed model (GLMM) to investigate the impact of system type on alpha diversity while accommodating repeated measures within each animal. The Cattle ID number was included as a random intercept to account for variability between cows. GLMM was chosen due to their ability to handle non-normal data distributions and incorporate both fixed and random effects (33). The model was implemented using glmer function from the lme4 package in R. We chose the analysis of compositions of microbiome with bias correction (ANCOM-BC) as our differential abundance (DA) analysis method for its competence in controlling the false discovery rate handling and applicability for longitudinal studies with repeated measures (34). DA analysis is conducted to determine taxa or ARG types that are differentially present between two or more environments such as sampling time points or systems (35). Procrustes analyses were used to evaluate the correlation between the cattle gastrointestinal resistome and taxonomic profiles (36, 37).

## RESULTS

### Characteristics of system A and B cattle

The average birth weight of system A calves was 67.5 lb, range 47–85 lb. System B calves averaged 74 lb, range 40–98 lb at birth. Sampling dates and summary characteristics of the cattle in the two systems are in Table 1. System A cattle averaged 639 lb and 22.8 months of age at the pre-harvest sampling (S5). In contrast, system B cattle averaged 1,173 lb and 12.4 months of age at the pre-harvest sampling (S5) (Table 1; Fig. S1). These data highlight a key difference between the two systems: pasture-raised grass-fed cattle take longer to gain weight and are generally harvested at an older age than conventionally raised grain-fed cattle.

**TABLE 1:** Sampling dates and cattle characteristics (age, weight) by production system

	S1	S2	S3	S4	S5
System A					
Sampling diet	Pre-weaning	Post-weaning	Summer	Winter	Pre-harvest
No.	33	32^*a*^	32^*a*^	33	30^*b*^
Sampling date^*d*^	05/03/2021	05/24/2021	08/05/2021	12/01/2021	07/12/2022
Age days (range)	259 (170, 414)	280 (191, 435)	353 (264, 508)	471 (382, 626)	694 (605, 849)
Weight lb (range)	416 (234, 558)	460 (248, 598)	448 (236, 616)	517 (254, 704)	639 (392, 840)
System B					
Sampling diet	Pre-weaning	Transition	Backgrounding	Finishing	Pre-harvest
No.	34	34	33^*c*^	33	33
Sampling date^*d*^	07/28/2021	09/22/2021	11/17/2021	01/12/2022	04/05/2022
Age days (range)	126 (587, 146)	182 (143, 202)	238 (199, 258)	294 (255, 314)	377 (338, 397)
Weight lb (range)	353 (260, 466)	431 (302, 540)	623 (484, 762)	831 (678, 988)	1173 (996,1355)

^*a*^ Two separate samples (periods S2 and S3) from one animal (H505) were not analyzed due to laboratory/sequencing error.

^*b*^ One death and two cattle sold in system A prior to pre-harvest sampling period.

^*c*^ One steer died between the second and third sampling periods.

^*d*^ Sampling dates are given as month/day/year.

### Diversity of ARGs

**Fig 1:**
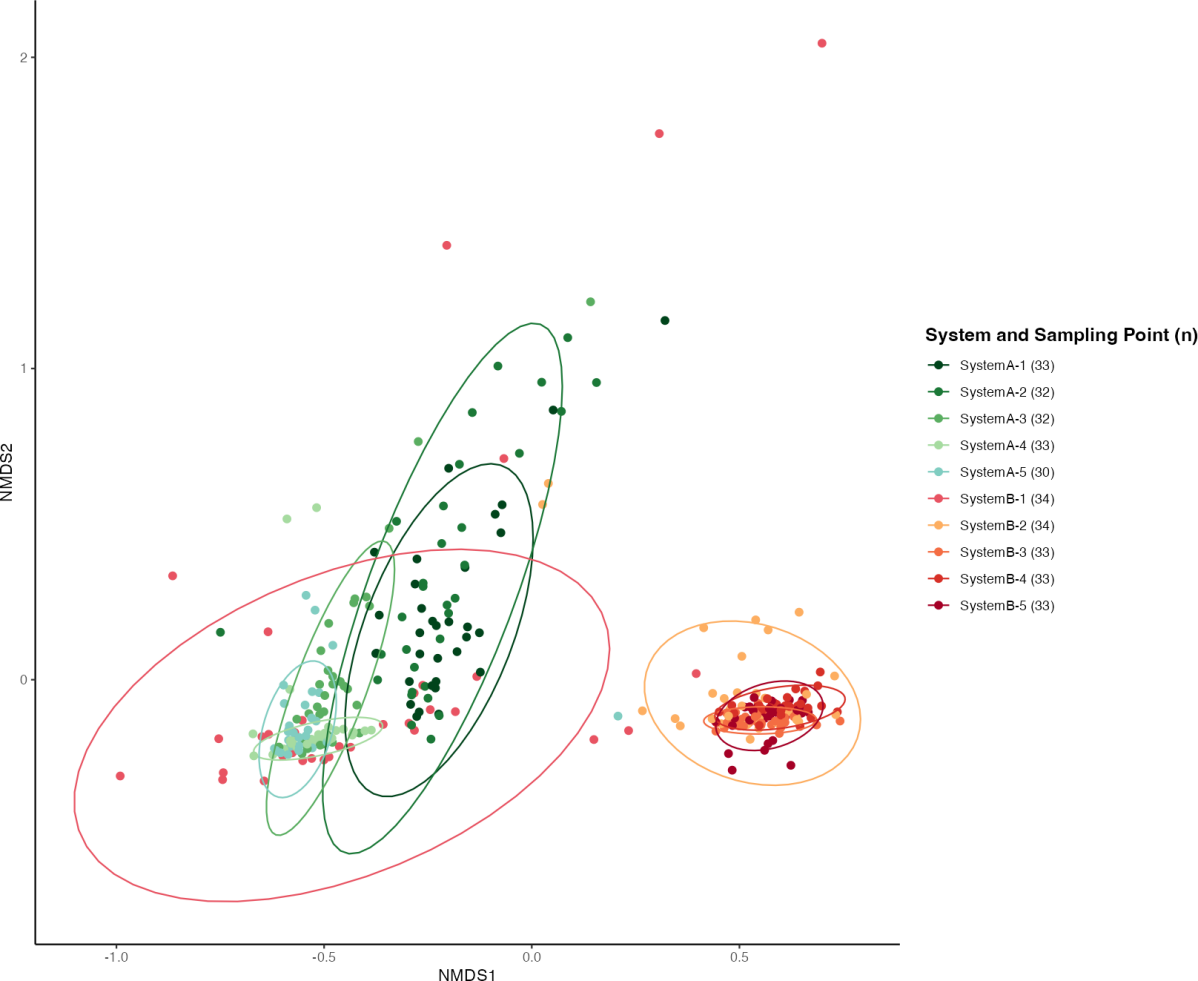
Beta-diversity ordination plot of type-level antibiotic resistance genes by system and sampling point. Non-metric multi-dimensional scaling (NMDS) plot is based on Bray–Curtis dissimilarities for *n* = 327 samples. The dispersion of each sampling point between two systems is represented by colored ellipses. Clustering by the system was confirmed using PERMANOVA (*P* < 0.001; *R*^2^ = 0.420; *n* = 4,000 permutations).

Shotgun metagenomic sequencing provided a non-discriminant, culture-independent, and high-resolution method to assess the community of ARGs (i.e., the resistome) within the two systems (38). As an initial step, we examined global compositional differences in the resistome by system and by sampling period. Bray–Curtis dissimilarity indices, which are measures of beta-diversity, were calculated, and NMDS ordination plots were used to visualize the differences in the community of ARGs in systems A and B (Fig. 1). These ordination plots indicate strong segregation of ARG communities by system (PERMANOVA, *P* ≤ 0.001; *R*^2^ = 0.420). However, system B pre-weaning (S1) samples overlap with all system A samples (S1–S5). Of note, the system B pre-weaning samples were taken during the period when cattle in both systems were milking and on pasture. The observed differences by system were likely due to differences in the compositional profile of ARGs rather than due to heterogeneity in variances (multivariate homogeneity of group dispersions, *P* = 0.524). The ordination plots also suggest segregation of ARG communities by sampling period (PERMANOVA *P* ≤ 0.001; *R*^2^ = 0.113). However, for the sampling periods, there was significant heterogeneity in variance (multivariate homogeneity of group dispersions, *P* = 0.007). Therefore, caution is warranted when interpreting the PERMANOVA sampling period results, as the observed differences in species composition may be due to either real differences between groups or may be influenced by differences in variability within each group. Altogether, differences in the system and sampling period alone captured a substantial overall variation across samples (*R*^2^ = 0.720).

**Fig 2:**
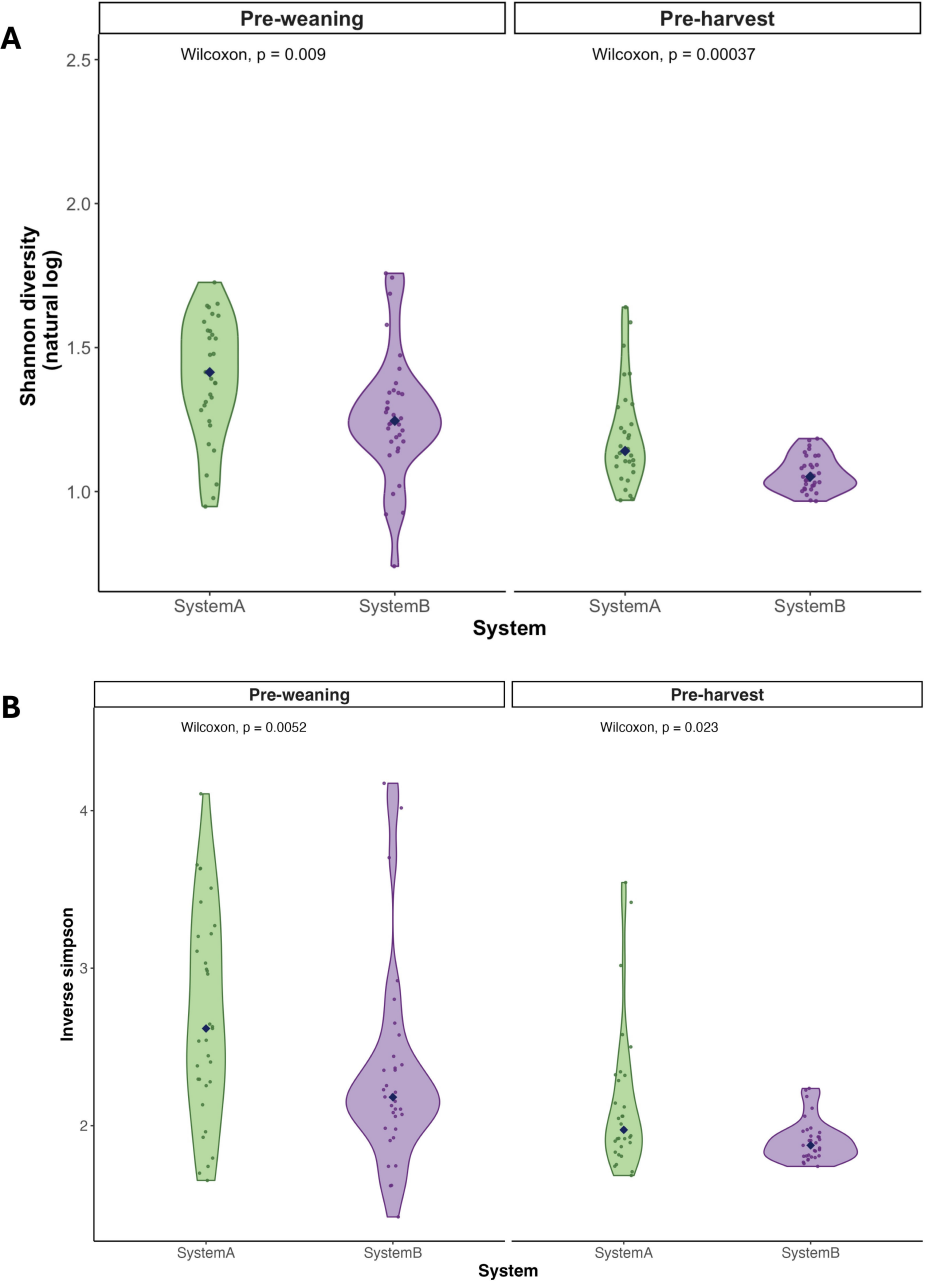
Alpha-diversity of antibiotic resistance genes at the type level. Violin plots compare alpha diversity indices between systems at pre-weaning (*n* = 33 system A and *n* = 34 system B samples) and pre-harvest (*n* = 30 system A and *n* = 33 system B samples). (**A**) Shannon (natural log) diversity and (**B**) inverse Simpson. Each jittered point represents an individual sample, and the median value for each group is indicated by a diamond in the center. Comparisons were made using a two-sided Wilcoxon rank-sum test.

Next, we compared alpha diversities by system at pre-weaning (S1) and pre-harvest (S5). Fig. 2A shows median Shannon diversity of type-level ARGs, and Fig. 2B shows inverse Simpson diversity of type-level ARGs. Alpha diversity was significantly higher in system A compared to system B at pre-weaning and pre-harvest for both indices. When comparing pre-weaning and pre-harvest samples, the observed effect size for Shannon diversity was 1.03 (Cohen’s *d* [*d*], 95% confidence interval [CI]: 0.49, 1.56) for system A and 1.25 (95% CI: 0.72, 1.78) for system B. The observed effect size for Inverse Simpson was 0.96 (95% CI: 0.43, 1.50) for system A and 0.91 (95% CI: 0.40, 1.42) for system B. Thus, there were large changes in alpha diversities between pre-weaning and pre-harvest samples in both systems.

We also investigated temporal trends in alpha diversity in each system in samples S1–S5; Shannon and inverse Simpson diversity trends are shown in Fig. S2A and B, respectively. In system A, alpha diversity was high at pre-weaning (S1), increased post-weaning (S2), and then generally declined over time. In system B, alpha diversity was highest at pre-weaning (S1), and then declined and leveled off. The median and range of alpha diversity indices by system and sample are in Table S5. The GLMM model indicated that system A cattle consistently experienced higher alpha diversity in the resistome over the course of their lifespan when compared to system B cattle ($\beta_{1}$ = −0.13, *P* = 0.008). The relationship between system and alpha diversity did not differ across different time points.

### Abundance of ARGs

**Fig 3:**
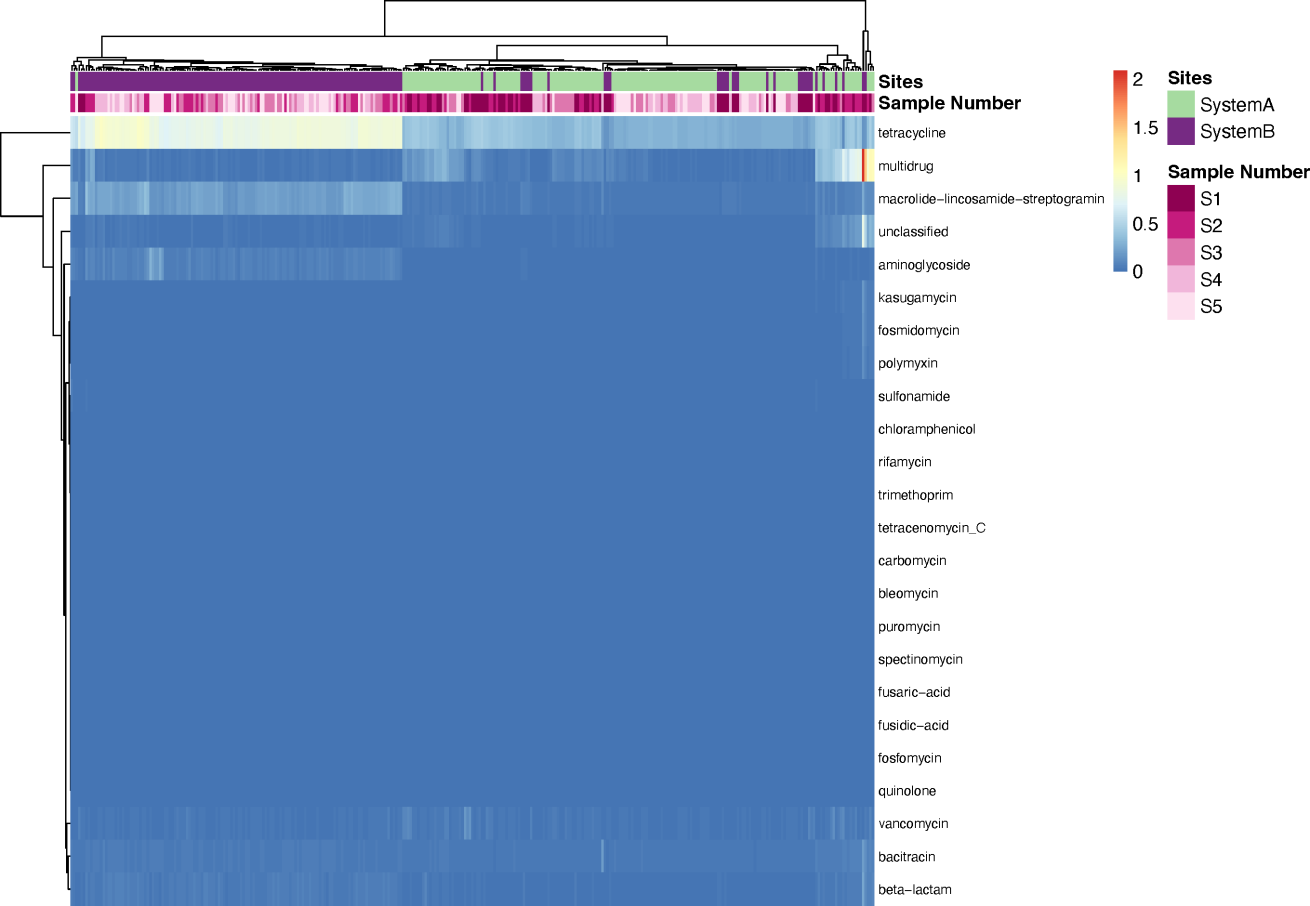
Heatmap of type-level antibiotic resistance gene (ARG) abundance. Data are log2 transformed for better visualization. Complete linkage clustering of 327 samples was based on ARG abundance. Bars represent the production system and sample number; the color key is indicated in the upper right corner.

**Fig 4:**
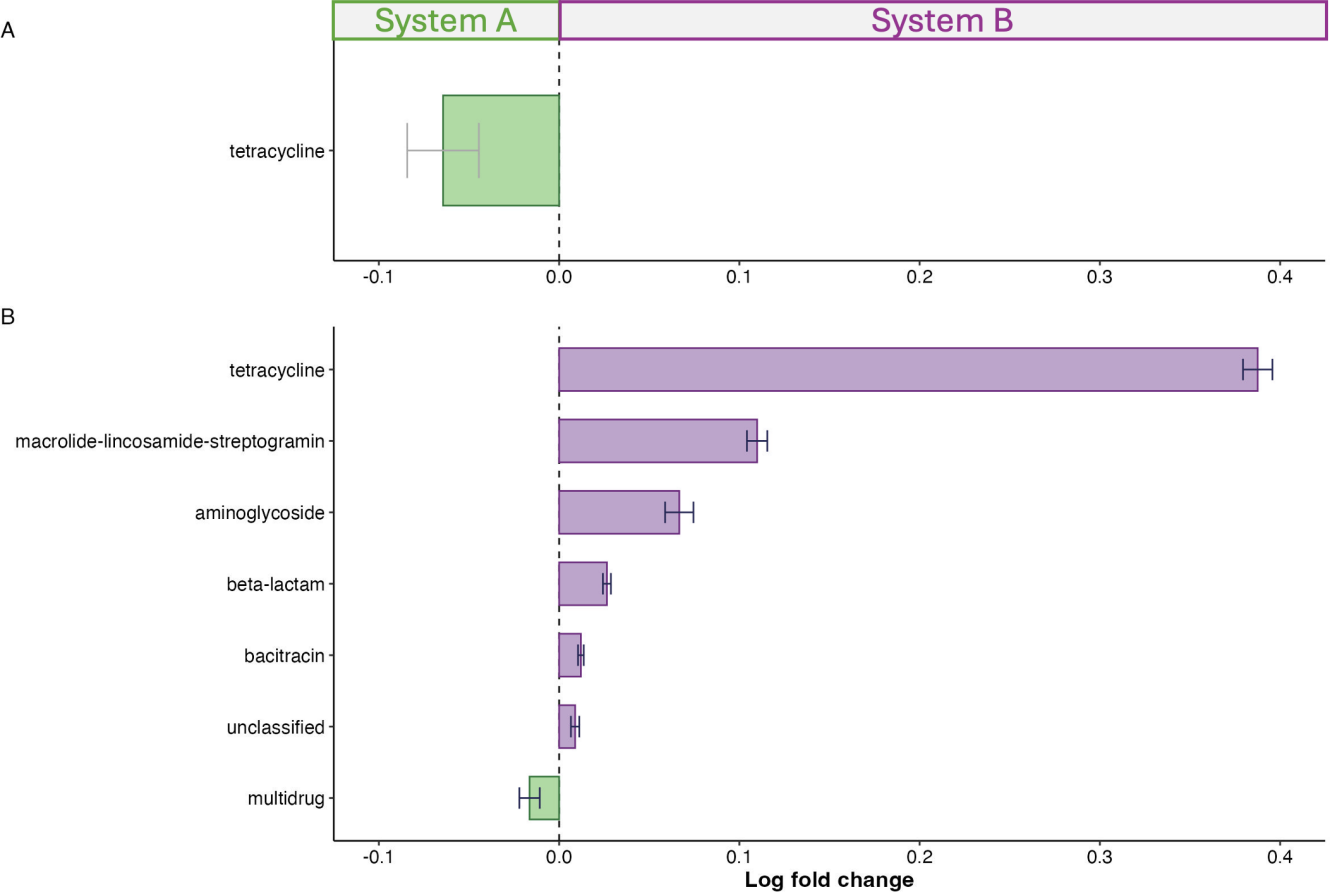
Differential abundance analysis of type-level antibiotic resistance genes (ARGs) in two systems. Analysis of compositions of microbiomes with bias correction was used to compare the relative abundance of type-level ARGs between the two systems at two stages: (**A**) pre-weaning and (**B**) pre-harvest. Purple markers indicate a higher abundance of ARGs in system B, and green bars indicate a higher abundance of ARGs in system A.

The analyses described above demonstrated that the beta and alpha diversities of ARGs differed between the two systems; additional analyses centered on specific resistance mechanisms. For visualization of the ARG types, we created a heatmap of log_2_-transformed relative abundance of the type-level ARGs, as shown in Fig. 3. The heatmap indicated high relative abundances of genes associated with resistance for tetracycline, multi-drug, and MLS antibiotics. The heatmap data were consistent with NMDS ordination plots of Bray–Curtis beta-diversity. Specifically, samples largely clustered by system, but several pre-weaning (S1) samples from system B cattle clustered with system A samples. ANCOM-BC analysis (35) was used to evaluate whether there were differences in the relative abundance of ARGs by type at pre-weaning (S1) and pre-harvest (S5). Tetracycline resistance genes were the only type of ARGs that differed in abundance in pre-weaning samples. As shown in Fig. 4A, the abundance of tetracycline ARGs was higher in system A cattle than in system B pre-weaning. At pre-harvest, tetracycline resistance was the biggest factor in differentiating these two systems, and levels were higher in system B (Fig. 4B). Levels of MLS, aminoglycosides, beta-lactam, bacitracin, and unclassified ARGs were also significantly higher in system B pre-harvest. In contrast, multidrug efflux ARGs were higher in system A pre-harvest (Fig. 4B).

Next, we examined temporal trends in ARGs that differed in abundance by system as determined in our ANCOM-BC analysis. The abundances of tetracycline, MLS, aminoglycosides, beta-lactam, bacitracin, and multidrug ARGs are shown in Fig. S3. In general, the relative abundances of tetracycline, MLS, aminoglycoside, beta-lactam, and bacitracin ARGs increased in system B post-weaning (S2) and remained higher than in system A in subsequent sampling periods when system B cattle were on grain-based diets.

We were also interested in examining the specific ARG subtypes. There were 367 unique ARG subtypes that were identified at least once among the 327 samples. Samples had a median of 109 ARG subtypes, range (52–198), and mean 110 standard deviation (SD) ± 19.14. System A samples had a median of 101 ARG subtypes, range (63–136), and mean 101 SD ± 12.8. System B samples had a median of 117 ARG subtypes, range (52–198), mean 118 SD ± 20.7. Many of these ARGs were infrequently detected. Therefore, we conducted prevalence filtering on the samples. The remaining 98 subtype-level ARGs are shown in a heatmap in Fig. S4. The three most abundant ARG subtypes encoded tetracycline resistance (*tetW* and *tetQ*) and MLS resistance (*mefA*).

### Taxonomic characterization of the fecal microbiota

**Fig 5:**
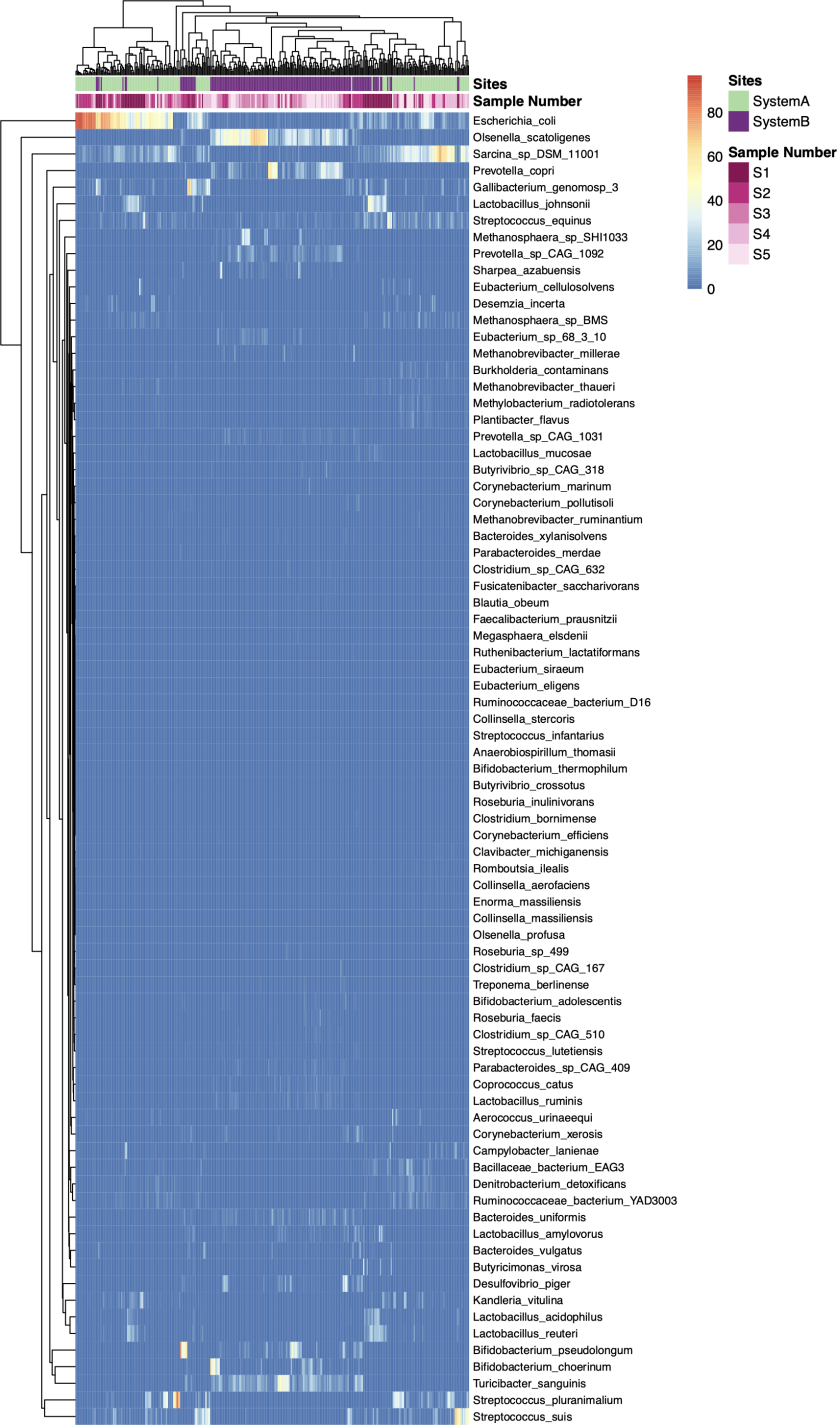
Taxonomic profile heatmap. Comparison of the log_2_-transformed abundances of 79 taxa across all samples (*n* = 327). Complete linkage clustering of samples was based on taxon abundance. Bars represent the production system and sample number; the color key is indicated in the upper right corner.

Taxonomic classiﬁcation of the shotgun metagenomic sequence data resulted in the identiﬁcation of 329 unique bacteria species that were present in at least one sample. Seventy-nine taxa remained after prevalence and variance filtering, and the samples largely clustered by site and sampling time (Fig. 5). There were eight species present in cattle in both systems, which were: *Olsenella scatoligenes*, *E. coli*, *Gallibacterium genomosp* 3, *Streptococcus equinus*, *Lactobacillus johnsonii*, *Sarcina* sp., *Streptococcus suis*, and *Lactobacillus reuteri. Turicibacter sanguinis* and *Prevotella copri* were the two most abundant taxa detected in system B pre-harvest (S5) samples. *E. coli* and *Sarcina* sp. were the most abundant bacterial species in system A pre-harvest (S5) samples. Potential human pathogens, such as *Salmonella enterica*, *Enterococcus faecali*s, and *C. difficile*, were not detected by shotgun metagenomic sequencing in any cattle in either system.

An NMDS plot of Bray–Curtis microbiota dissimilarity by system is shown in Fig. S5. These data suggest that the bacterial communities significantly differed in system A and B cattle (*P* < 0.001). However, pre-weaning samples (S1) from system B cattle were distinct from the rest of the system B samples and overlapped with system A samples. Moreover, there was a clear distinction between system A and the remaining system B samples (S2–S5). These data were similar to trends identified in the ARG data.

### Correlations between the resistome and microbiota

**Fig 6:**
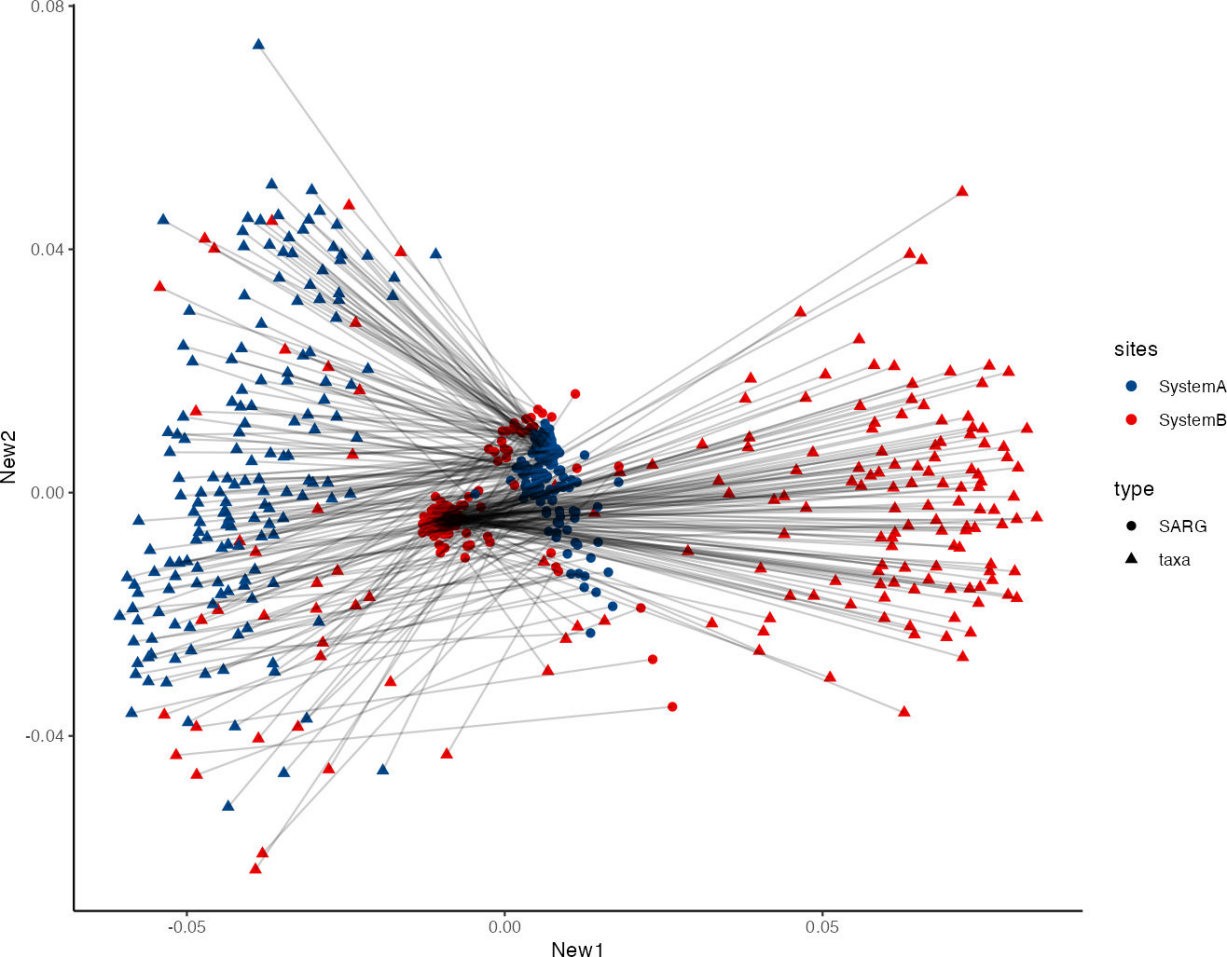
Procrustes analysis results of the antibiotic resistance genes (ARG) and microbial taxa community. Each point represents either the individual sample’s taxonomic community or the ARG community (*n* = 327 samples). Community structures are marked by different shapes, ARG (circle), and taxa (triangle). Samples are colored by the system, systems A (blue) and B (red). Lines connect individual sample’s corresponding taxa and ARG community. A significant correlation between two ordination plots was confirmed using the protest function in the “vegan” package in R (*M*^2^ = 0.958; *P* = 0.001; *n* = 999 permutations).

The prior analyses indicated that there were similar patterns of change in the resistome and microbiota in the two systems. Thus, we hypothesized that changes in the resistome were driven by changes in composition of the bacterial community. Procrustes analysis (36, 37) visualized in Fig. 6 confirmed a significant correlation between ARG and bacterial taxonomic community profiles in samples from systems A and B (*M*^2^ = 0.958; *P* = 0.001). The figure colored by system illustrates how relative differences in ARG composition (circles) correlate with bacterial composition (triangles) differences between the two systems. Thus, this analysis highlights the interdependence of the ARG and bacterial community structures. The temporal trend of this matrix correlation over five sampling periods is shown in Fig. S6. In most sampling points, the pattern mirrors the main figure, where relative differences in ARG compositions correlate with divergence in distinct bacterial communities between the systems. However, sampling period 1 deviates from this trend; the ARGs and bacterial communities are similar when comparing the two systems, all located to the left of the figure.

## DISCUSSION

The majority of antibiotics used in the US and throughout the world are used in agriculture (5). This has led to One Health concerns that antimicrobial use in livestock can exacerbate antibiotic resistance. The extent to which this happens is unknown, and additional research is needed to support judicious antibiotic use for animal, human, and environmental health (2, 18, 39). We conducted a study to compare the abundance and type of ARGs and bacterial species in fecal samples from pasture-raised grass-fed and conventionally raised grain-fed cattle. Beta-diversity measures indicated that the pre-weaning (S1) communities of ARGs and bacterial taxa were relatively similar in the two systems; these samples were taken when cattle in both systems were milking and on pasture. The ARG and taxonomic communities in systems A and B became more dissimilar after weaning and coinciding with the transition of system B cattle to a grain-based diet with feed additives containing ionophores. Diversity of ARGs was higher in system A than in system B across the cattle lifespan. However, higher relative abundances of ARGs were observed post-weaning in system B compared to system A for aminoglycoside, MLS, beta-lactam, and tetracycline antibiotics.

Our data are consistent with other studies of the cattle resistome, reviewed by Haley and Kessel (38, 39) and Ma et. al (40), which have consistently shown that the cattle fecal resistome is dominated by ARGs for tetracycline and MLS antibiotics. ARGs for beta-lactams and aminoglycosides are also frequently detected (38, 40). Noyes et al. observed a decrease in resistome diversity when cattle were in feedlots and a high prevalence of tetracycline and MLS ARGs when cattle left the feedlots for slaughter (17). Macrolides (e.g., tylosin) and tetracyclines (e.g., chlortetracycline) are often administered in feed for relatively long durations to reduce liver abscesses (18). Aminoglycoside use in U.S. beef cattle production is discouraged and thought to be low due to the extended withdrawal times and potential for high residue levels in meat (18). With the exception of one steer in system B that received a single dose of a macrolide, medically important antibiotics were not provided to cattle in either system. The persistence of ARGs in both systems, even in the absence of medically important antibiotic use and the relatively high levels of tetracycline, MLS, and other ARGs in post-weaning system B samples, indicates that factors other than medically important antimicrobial use contribute to differences in the resistome. The differences may be related to diet and ionophore use in system B, low fitness costs associated with specific resistance mechanisms, environmental contamination with antibiotics, and/or co-selection of antibiotic resistance linked to other genetic traits, such as genes encoding for resistance to heavy metals (41–44).

The overlap in resistomes and microbiota in pre-weaning samples from both systems and the increase in the relative abundance of ARGs as system B cattle shift to grain-based diets are notable. Collectively, our data suggest that diet has an impact on resistance levels and the microbiota. Diet could potentially influence the abundance and diversity of ARGs by affecting factors like nutrient availability, oxygen levels, and pH, which in turn shape the gut microbiota. The specific taxa within the microbiota may vary in their propensity to carry ARGs (42, 45, 46). Liu et al. demonstrated variation by diet in the fecal resistome and microbiota of dairy cows (42). This study indicated that colostrum was a source of ARGs in calves, and that increasing levels of fiber in the diet were associated with lower levels of ARGs (42). Additional research in dairy cattle demonstrated that dietary changes resulted in altered metabolic pathways and lower abundances of fiber-degrading taxa when cattle shifted from forage to grain (47). Auffret et al. examined the rumen microbiota and resistome and found higher levels of Proteobacteria and aminoglycoside ARGs in concentrate-fed cattle compared to forage-fed cattle (48). Additionally, the use of heavy metals and biocides in feed may co-select for ARGs (42, 44). The cattle in system B had metals, such as copper sulfate and zinc sulfate, in their feed (see Tables S1 to S3). While we did not detect human or zoonotic pathogens by shotgun metagenomic sequencing, others have shown that high-grain diets contribute to increased prevalence and shedding of pathogens, such as enterohemorrhagic *E. coli* O157, in beef cattle (49). Thus, farm management practices, such as providing diets that contain high levels of fiber, may help lower the abundance of ARGs in the bovine microbiota.

Approximately 54% of antibiotics used in animal agriculture in the United States are considered medically important, and ≥13 antibiotics are approved by the FDA for use in feed for disease prevention without explicit instructions for the duration of their use (50). Ionophores are the second most common antibiotic class that is used in animals in the United States; they are considered less relevant to human health because they are used exclusively in animals (16). While not considered medically important, their use has the potential to carry additional risk related to co-selection (i.e., when resistance genes are genetically linked) and/or cross-resistance (i.e., a mechanism or mutation that confers resistance to two or more drugs) (16). System B cattle received Rumensin monensin, which is an ionophore isolated from *Streptomyces cinnamonensis* that targets gram-positive bacteria (51). Our data suggest that ionophores may contribute to the higher levels of medically important ARGs observed in system B cattle. A few other studies have examined the impact of ionophore use on antibiotic resistance (16). Monensin has been associated with increased macrolide resistance in *Enterococcus* isolates from cattle (52). Thomas et al. compared five treated and five control steer and examined the impact of feed additives containing monensin and tylosin on the gastrointestinal tract resistome and microbiota. They did not identify significant differences in the abundance of ARGs (19). Additional large studies that directly compare cattle on the same diet, with and without ionophores, are needed to definitively address the role of ionophores on resistance to medically important antibiotics.

There were several limitations in our study. We compared two different production systems. In addition to diet, several other factors differed in the two systems, including breed, location, timing of sampling, and lifespan (45). These dissimilarities are reflective of real-world differences in cattle production systems and are also a general challenge in the field since breed, management practices, and diets frequently vary based on factors that include production goals, location, and cost (38). The bovine microbiome has been shown to vary by sex, breed, and host genetics (45, 53). We were unable to disentangle the effect of diet from other factors, such as host-genetics, sex, and ionophore use, that likely contribute to variations in ARGs and the microbiota. Further studies are warranted that compare calves of the same sex and genetic background on a single farm. However, this would be logistically challenging, and regenerative grass-fed and grain systems each have unique infrastructure, resource needs, and management practices that do not usually coexist within a single farm’s operational model. We obtained data on vaccines, and both study sites provided vaccines for respiratory viruses, diarrhea, and *Clostridium* species. Few, if any, studies have investigated whether vaccine administrations are associated with changes in the bovine microbiota or resistome (54). We did not attempt to examine the impact of vaccines on the microbiota and resistome, as this was beyond the scope of the current study.

A strength of these data is that we prospectively followed individual cattle from pre-weaning to harvest. We demonstrated that system B grain-fed cattle have comparatively higher levels of ARGs for tetracycline, MLS, aminoglycoside, and beta-lactam antibiotics in comparison to system A grass-fed cattle. These data support the contention that specific farm management practices may provide a path to reduce antimicrobial resistance. However, the pasture-raised cattle in system A cattle take longer to reach market weight and weigh less at harvest. Vikram et al. hypothesized that slightly lower abundances of ARGs observed in cattle raised without antibiotics may not significantly affect levels of resistance in the environment if the additional manure from their longer lifespans is considered (18). These data also demonstrate the complexity of antimicrobial resistance, and that reductions in the use of antibiotics in agriculture will have to be part of a multifaceted approach to reduce resistance. The increasing veterinary and medical challenges that arise from the emergence of antibiotic resistance need to be considered along with the ethical treatment of animals and the need to sustainably and affordably feed the human population. Future studies should focus on the development of risk and ranking models for ARGs and consider the impact of ionophore use on the microbiota and resistome.

## Data Availability

The metadata that support the findings of this study are available from the corresponding author upon request. The shotgun metagenomic sequencing reads have been deposited with the NCBI Sequence Read Archive and are available under BioProject PRJNA1056763.

## SUPPLEMENTAL MATERIAL

Supplemental Figures msphere.00738-24-s0001.docx Fig. S1 to S6.

Supplemental Tables msphere.00738-24-s0002.docx Tables S1 through S5.

ASM does not own the copyrights to Supplemental Material that may be linked to, or accessed through, an article. The authors have granted ASM a non-exclusive, world-wide license to publish the Supplemental Material files. Please contact the corresponding author directly for reuse.
