# Supplementary material for: Prospective comparison of the digestive tract resistome and microbiota in cattle raised in grass-fed versus grain-fed production systems
Source: mSphere. 2025 Feb 14;10(3):e00738-24. doi: 10.1128/msphere.00738-24 (PMC11934311; doi:10.1128/msphere.00738-24)

**Prospective comparison of the digestive tract resistome and microbiota in cattle raised in grass-fed versus grain-fed production systems**

**SUPPLEMENTAL FIGURES**

**Supplemental Figure 1. Fecal swab sampling scheme by system.** Sampling scheme timeline for cattle by system. The colored lines represent the duration of the life cycle of cattle in each system, green for system A and orange for system B. Each dot represents a sampling point and the colors of the dot represent diet (grass vs grain).

**Supplemental Figure 2. Alpha diversity of type-level antibiotic resistance genes (ARGs) across five different sampling periods by system.** (A) Shannon (natural log) diversity and (B) inverse Simpson. Green and purple jitter points represent individual samples from systems A and B, respectively. Triangles and dots represent the median ARG diversity at each sampling point.

**Supplemental Figure 3. Temporal trends in select antibiotic resistance genes (ARGs) stratified by system.** ARG abundance at five different sampling periods. Green and purple jitter points represent individual samples for system A and B, respectively. Triangles and dots represent the median ARG abundance at each sampling point.

**Supplemental Figure 4. Heatmap of subtype-level antibiotic resistance genes (ARGs) abundance.** Data are log2 transformed for visualization. Complete linkage clustering of samples (n=327) was based on subtype-level ARG abundance. Bars represent the production system and sample number; the color key is indicated in the upper right corner.

**Supplemental Figure 5. Beta-diversity ordination plot of bovine gastrointestinal microbiota profile by system and sampling point.** Non-metric Multi-dimensional Scaling (NMDS) plot is based on Bray-Curtis dissimilarities for n = 327 samples. The dispersion of each sampling point between two systems is represented by colored ellipses. Clustering by the system was confirmed using PERMANOVA (P < 0.001; n = 4000 permutations).


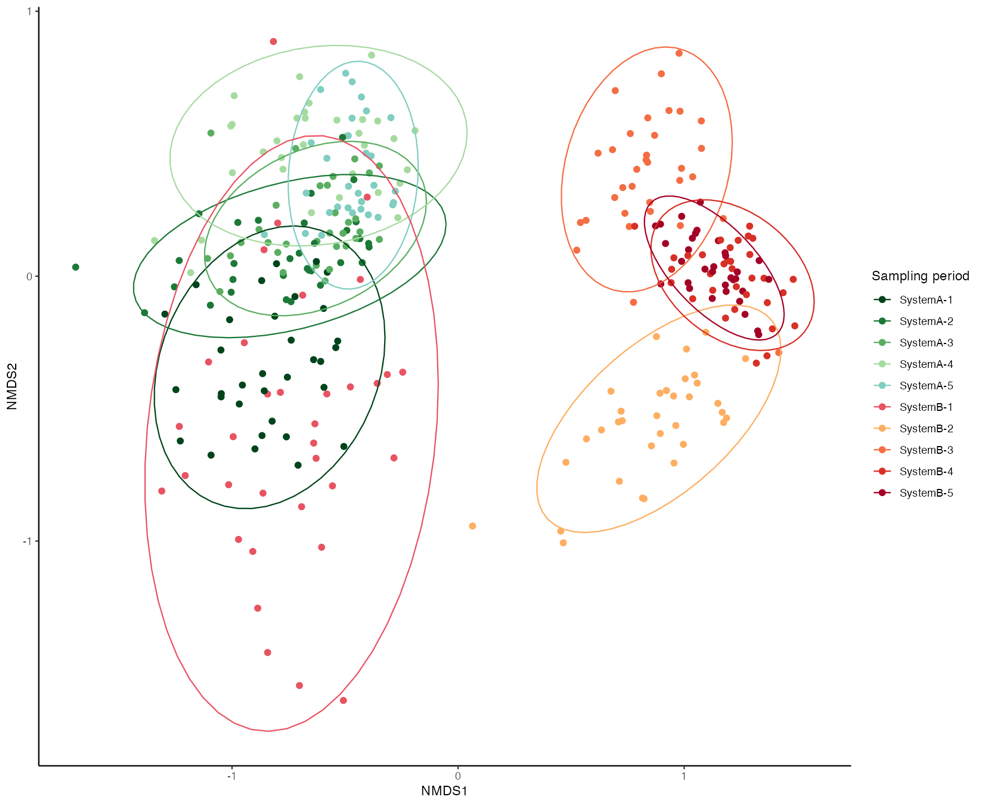


**Supplemental Figure 6. Procrustes analysis results of the antibiotic resistance genes (ARG) and microbial taxa community stratified by sampling point.** Each point represents either the individual sample’s taxonomic community or the ARG community. Community structures are marked by different shapes, ARG (circle) and taxa (triangle). Samples are colored by the system, System A (blue) and System B (red). Lines connect individual sample’s corresponding taxa and ARG community.


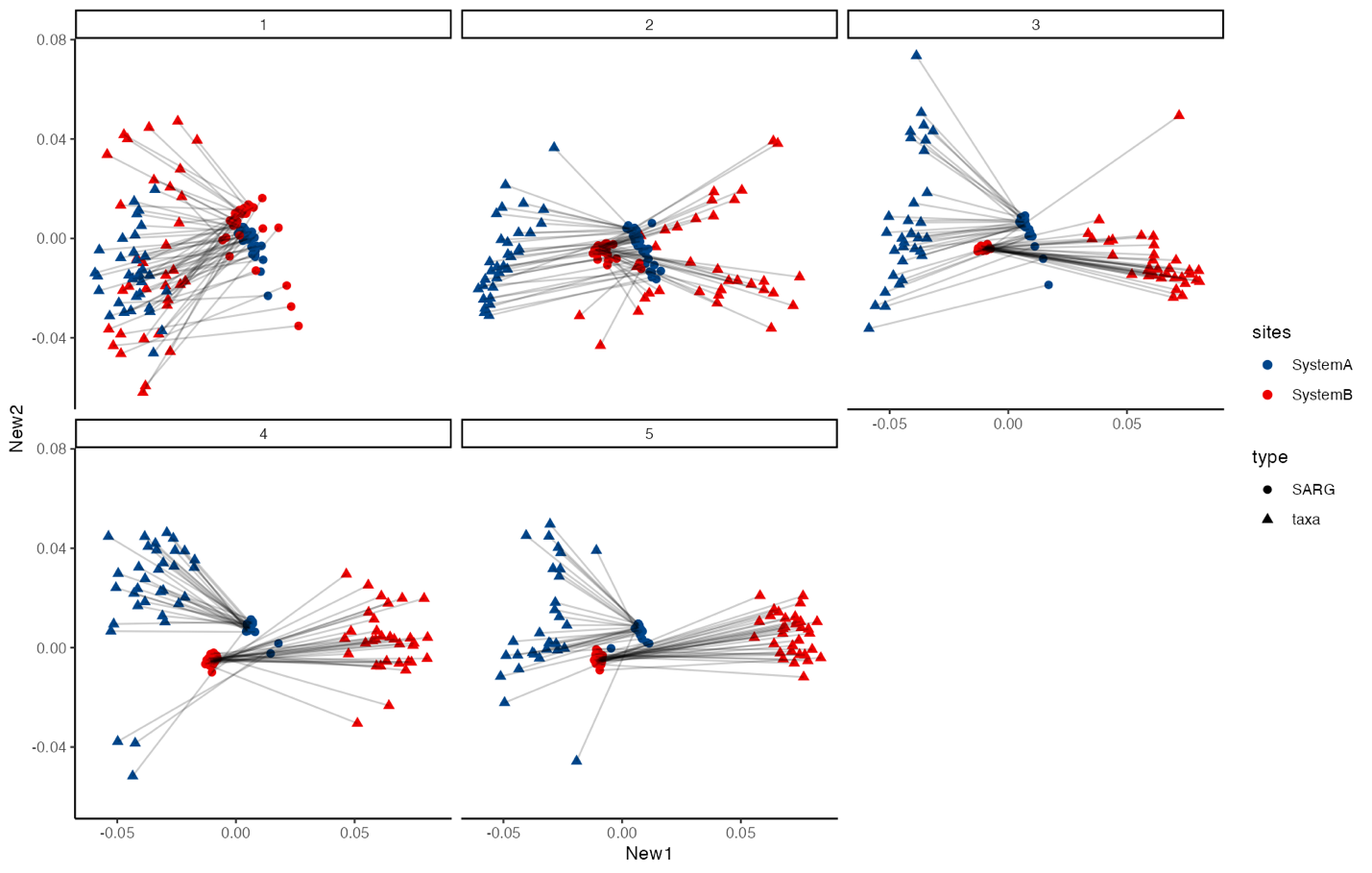

Supplement: Supplemental Figures — Fig. S1 to S6. [file msphere.00738-24-s0001.docx]
