# Supplementary material for: Prospective comparison of the digestive tract resistome and microbiota in cattle raised in grass-fed versus grain-fed production systems
Source: mSphere. 2025 Feb 14;10(3):e00738-24. doi: 10.1128/msphere.00738-24 (PMC11934311; doi:10.1128/msphere.00738-24)
Supplement: Supplemental Tables — Tables S1 through S5. [file msphere.00738-24-s0002.docx]

**Supplemental Table 1.** System B transition diet

|  |  | **Low grain diet (LG)** | | **High grain diet (HG)** | |
| --- | --- | --- | --- | --- | --- |
| **Type** | **Ingredient** | **Percentage** | **Pounds or grams** | **Percentage** | **Pounds or grams** |
| Early weaning diet | Wole shelled corn | 38.043 | 380.43 | 67.441 | 674.41 |
|  | Soybean hulls (pelleted) | 9.782 | 97.82 | 0 | 0 |
|  | DDGS (pelleted) | 41.304 | 413.04 | 0 | 0 |
|  | B940 (pelleted) | 10.871 | 108.71 | 32.559 | 325.59 |
| Early weaning premix formula | Soybean hulls (pelleted) | 65.404 | 654.04 | 0 | 0 |
|  | Soybean Hulls | 0 | 0 | 14.286 | 142.86 |
|  | DDGS | 0 | 0 | 50 | 500 |
|  | Urea | 0 | 0 | 1.929 | 19.29 |
|  | Soybean Meal | 0 | 0 | 22.938 | 229.38 |
|  | Limestone | 16.593 | 165.93 | 4.646 | 46.46 |
|  | Salt | 4.609 | 46.09 | 1.291 | 12.91 |
|  | Vitamin A | 0.068 | 0.68 | 0.019 | 0.19 |
|  | Vitamin D-3 | 0.068 | 0.68 | 0.019 | 0.19 |
|  | Vitamin E | 0.205 | 2.05 | 0.057 | 0.57 |
|  | Calcium Sulfate | 6.453 | 64.53 | 1.807 | 18.07 |
|  | Selenium | 0.35 | 3.5 | 0.098 | 0.98 |
|  | Rumesin 90 | 0.157 | 1.57 | 0.043 | 0.43 |
|  | Potassium Chloride | 2.765 | 27.65 | 0.774 | 7.74 |
|  | Copper Sulfate | 0.055 | 0.55 | 0.015 | 0.15 |
|  | Zinc Sulfate | 0.184 | 1.84 | 0.052 | 0.52 |
|  | Manganese Sulfate | 0.088 | 0.88 | 0.025 | 0.25 |
|  | Cobalt Carbonate | 0.001 | 0.01 | 0.001 | 0.01 |
|  | AV Blend | 3 | 30 | 2 | 20 |

**Supplemental Table 2.** System B backgrounding diet

| **Type** | **Ingredient** | **Percentage** | **Pounds or grams** |
| --- | --- | --- | --- |
| Short backgrounding diet | Cracked corn | 33.75 | 337.5 |
|  | Soy Hulls (pelleted) | 37.5 | 375 |
|  | DDGS (pelleted) | 16.25 | 162.5 |
|  | B930 (pelleted) | 12.5 | 125 |
| Short backgrounding premix formula | Urea | 5.39 | 53.9 |
|  | Soybean Meal | 30.414 | 304.14 |
|  | Limestone | 16.593 | 165.93 |
|  | Salt | 4.609 | 46.09 |
|  | Vitamin A | 0.068 | 0.68 |
|  | Vitamin D-3 | 0.068 | 0.68 |
|  | Vitamin E | 0.205 | 2.05 |
|  | Calcium Sulfate | 6.453 | 64.53 |
|  | Selenium | 0.35 | 3.5 |
|  | Rumesin 90 | 0.157 | 1.57 |
|  | Potassium Chloride | 2.765 | 27.65 |
|  | Copper Sulfate | 0.055 | 0.55 |
|  | Zinc Sulfate | 0.184 | 1.84 |
|  | Manganese Sulfate | 0.088 | 0.88 |
|  | Cobalt Carbonate | 0.001 | 0.01 |
|  | AV Blend | 3 | 30 |
|  | Ground Corn | 29.6 | 296 |

**Supplemental Table 3.** System B finishing diet

| **Type** | **Ingredient** | **Percentage** | **Pounds or grams** |
| --- | --- | --- | --- |
| Finishing diet | Cracked corn | 69.565 | 695.65 |
|  | DDGS (pelleted) | 19.565 | 195.65 |
|  | B944 (pelleted) | 10.87 | 108.7 |
|  |  |  |  |
| Finishing diet premix formula | Urea | 3.692 | 36.92 |
|  | Limestone | 16.614 | 166.14 |
|  | Salt | 4.615 | 46.15 |
|  | Vitamin A | 0.068 | 0.68 |
|  | Vitamin D-3 | 0.068 | 0.68 |
|  | Vitamin E | 0.205 | 2.05 |
|  | Calcium Sulfate | 6.461 | 64.61 |
|  | Selenium | 0.351 | 3.51 |
|  | Rumesin 90 | 0.203 | 2.03 |
|  | Potassium Chloride | 2.769 | 27.69 |
|  | Copper Sulfate | 0.06 | 0.6 |
|  | Zinc Sulfate | 0.185 | 1.85 |
|  | Manganese Sulfate | 0.111 | 1.11 |
|  | Cobalt Carbonate | 0.001 | 0.01 |
|  | AV Blend | 3 | 30 |
|  | Ground Corn | 61.597 | 615.97 |

**Supplemental Table 4.** Sequencing summary statistics, n=327 samples

| **BarcodeID** | **Total pairs** | **Trimmed**  **pairs** | **Classified** | **Unclassified** | **Sampling number** | **System** |
| --- | --- | --- | --- | --- | --- | --- |
| 210181 | 44673624 | 42887284 | 5021193 | 37866091 | S1 | B |
| 210182 | 38663173 | 37212015 | 6501753 | 30710262 | S2 | B |
| 210281 | 36976346 | 35500001 | 5145513 | 30354488 | S1 | B |
| 210282 | 40510653 | 39023031 | 8239257 | 30783774 | S2 | B |
| 210381 | 36165616 | 34799050 | 8374928 | 26424122 | S1 | B |
| 210382 | 42430463 | 40667197 | 8539927 | 32127270 | S2 | B |
| 210481 | 63271580 | 59942291 | 7941060 | 52001231 | S1 | B |
| 210482 | 40870717 | 39409948 | 7208509 | 32201439 | S2 | B |
| 210581 | 53067575 | 49689322 | 6429748 | 43259574 | S1 | B |
| 210582 | 41914377 | 40347482 | 7719846 | 32627636 | S2 | B |
| 210681 | 49933546 | 47815460 | 7482835 | 40332625 | S1 | B |
| 210682 | 33493283 | 32595679 | 7870151 | 24725528 | S2 | B |
| 210781 | 51124517 | 48921406 | 6764889 | 42156517 | S1 | B |
| 210782 | 30546429 | 29609908 | 4844630 | 24765278 | S2 | B |
| 210881 | 40040934 | 37980880 | 7284166 | 30696714 | S1 | B |
| 210882 | 28949228 | 28181918 | 4936366 | 23245552 | S2 | B |
| 210981 | 39390312 | 37528223 | 6462848 | 31065375 | S1 | B |
| 210982 | 32891655 | 31934140 | 5422960 | 26511180 | S2 | B |
| 211081 | 30000835 | 28706555 | 3814554 | 24892001 | S1 | B |
| 211082 | 33369013 | 32426790 | 4267144 | 28159646 | S2 | B |
| 211181 | 35777841 | 34088710 | 7184940 | 26903770 | S1 | B |
| 211182 | 32048734 | 31143377 | 6663439 | 24479938 | S2 | B |
| 211281 | 43714227 | 42000543 | 6517478 | 35483065 | S1 | B |
| 211282 | 31789689 | 30875814 | 6077734 | 24798080 | S2 | B |
| 211381 | 49307654 | 47009478 | 9108564 | 37900914 | S1 | B |
| 211382 | 33278034 | 32333679 | 5724303 | 26609376 | S2 | B |
| 211481 | 52930584 | 50988397 | 8280847 | 42707550 | S1 | B |
| 211482 | 45399355 | 44129372 | 7505327 | 36624045 | S2 | B |
| 211581 | 53645474 | 51651770 | 7373364 | 44278406 | S1 | B |
| 211582 | 43020641 | 41809136 | 8536895 | 33272241 | S2 | B |
| 211681 | 35867562 | 34435777 | 6636024 | 27799753 | S1 | B |
| 211682 | 54153509 | 52658739 | 7409763 | 45248976 | S2 | B |
| 211781 | 40158939 | 38443720 | 5434779 | 33008941 | S1 | B |
| 211782 | 42475855 | 41050754 | 7053587 | 33997167 | S2 | B |
| 211784 | 85398298 | 80415741 | 13879544 | 66536197 | S4 | B |
| 211881 | 40890187 | 39181839 | 9543127 | 29638712 | S1 | B |
| 211882 | 40621775 | 39472642 | 7322255 | 32150387 | S2 | B |
| 211981 | 40603152 | 38996173 | 5081918 | 33914255 | S1 | B |
| 211982 | 30575169 | 29727938 | 4438090 | 25289848 | S2 | B |
| 212081 | 47705333 | 45462899 | 8970858 | 36492041 | S1 | B |
| 212082 | 44319087 | 43058054 | 6683074 | 36374980 | S2 | B |
| 212181 | 51518281 | 49149338 | 10196544 | 38952794 | S1 | B |
| 212182 | 38421435 | 37330589 | 7884453 | 29446136 | S2 | B |
| 212281 | 50890829 | 48647844 | 7691634 | 40956210 | S1 | B |
| 212282 | 25958926 | 25172777 | 4021901 | 21150876 | S2 | B |
| 212381 | 40653673 | 38956084 | 5895271 | 33060813 | S1 | B |
| 212382 | 28219005 | 27243675 | 4490464 | 22753211 | S2 | B |
| 212481 | 43464006 | 41364817 | 6090317 | 35274500 | S1 | B |
| 212482 | 26817981 | 26051809 | 3591601 | 22460208 | S2 | B |
| 212581 | 38879055 | 36842295 | 5807247 | 31035048 | S1 | B |
| 212582 | 20652820 | 19937420 | 3058223 | 16879197 | S2 | B |
| 212681 | 34227668 | 32668578 | 6521710 | 26146868 | S1 | B |
| 212682 | 25539429 | 24673438 | 3545076 | 21128362 | S2 | B |
| 212781 | 51162841 | 49091252 | 7573024 | 41518228 | S1 | B |
| 212782 | 30481162 | 29517095 | 4430414 | 25086681 | S2 | B |
| 212881 | 40160555 | 38416654 | 7622321 | 30794333 | S1 | B |
| 212882 | 25931877 | 25134542 | 3987610 | 21146932 | S2 | B |
| 212981 | 53182824 | 50886932 | 11167457 | 39719475 | S1 | B |
| 212982 | 24876769 | 24002060 | 4521531 | 19480529 | S2 | B |
| 213081 | 42829868 | 41064510 | 6233355 | 34831155 | S1 | B |
| 213181 | 42863925 | 41004330 | 7412693 | 33591637 | S1 | B |
| 213281 | 41369173 | 39659634 | 5714364 | 33945270 | S1 | B |
| 213381 | 44160286 | 42068678 | 7771519 | 34297159 | S1 | B |
| 213481 | 40566739 | 39085190 | 5321397 | 33763793 | S1 | B |
| H38181 | 18138717 | 17273541 | 2995565 | 14277976 | S1 | A |
| H38182 | 17085092 | 16254238 | 3188285 | 13065953 | S2 | A |
| H38183 | 39873256 | 38465582 | 6075249 | 32390333 | S3 | A |
| H3818501 | 38382512 | 36472607 | 4995539 | 31477068 | S5 | A |
| H38381 | 15503392 | 14694586 | 2599763 | 12094823 | S1 | A |
| H38382 | 21393739 | 20223580 | 3181021 | 17042559 | S2 | A |
| H38383 | 36275715 | 34864639 | 5762359 | 29102280 | S3 | A |
| H3838501 | 35632023 | 33856772 | 4240933 | 29615839 | S5 | A |
| H38881 | 17628902 | 16694201 | 2987260 | 13706941 | S1 | A |
| H38882 | 21950146 | 20890419 | 3300631 | 17589788 | S2 | A |
| H38883 | 35932659 | 34513204 | 4775558 | 29737646 | S3 | A |
| H3888501 | 36264701 | 34219792 | 3843085 | 30376707 | S5 | A |
| H40581 | 18023820 | 17140435 | 2405525 | 14734910 | S1 | A |
| H40582 | 21075817 | 20014500 | 2884681 | 17129819 | S2 | A |
| H40583 | 35957683 | 34623474 | 5330443 | 29293031 | S3 | A |
| H4058501 | 40088303 | 38030474 | 4843419 | 33187055 | S5 | A |
| H40681 | 17587653 | 16729949 | 2699304 | 14030645 | S1 | A |
| H40682 | 22557032 | 21516316 | 4250759 | 17265557 | S2 | A |
| H40683 | 41788357 | 40163997 | 6199644 | 33964353 | S3 | A |
| H4068501 | 31945807 | 30095493 | 4439854 | 25655639 | S5 | A |
| H41081 | 20028997 | 19029835 | 2921266 | 16108569 | S1 | A |
| H41082 | 20216281 | 19253818 | 3965449 | 15288369 | S2 | A |
| H41083 | 38508777 | 37023420 | 5518124 | 31505296 | S3 | A |
| H4108501 | 32219069 | 30422196 | 4120194 | 26302002 | S5 | A |
| H41181 | 18204891 | 17238504 | 2741594 | 14496910 | S1 | A |
| H41182 | 21476421 | 20359754 | 3141702 | 17218052 | S2 | A |
| H41183 | 32770512 | 31471521 | 5677939 | 25793582 | S3 | A |
| H4118501 | 31473041 | 29610372 | 4261567 | 25348805 | S5 | A |
| H41881 | 18006342 | 16995860 | 2867068 | 14128792 | S1 | A |
| H41882 | 22204336 | 21033170 | 3291758 | 17741412 | S2 | A |
| H41883 | 41390017 | 39977444 | 6663257 | 33314187 | S3 | A |
| H41884 | 36108393 | 33839313 | 4265970 | 29573343 | S4 | A |
| H42281 | 16765926 | 15887057 | 2790228 | 13096829 | S1 | A |
| H42282 | 20012155 | 18982965 | 2992486 | 15990479 | S2 | A |
| H42283 | 40343301 | 38831757 | 5897234 | 32934523 | S3 | A |
| H4228501 | 24944758 | 23395529 | 3168251 | 20227278 | S5 | A |
| H44181 | 16940166 | 15988179 | 2522009 | 13466170 | S1 | A |
| H44182 | 20354445 | 19420571 | 3320325 | 16100246 | S2 | A |
| H44183 | 34437402 | 33089166 | 5165592 | 27923574 | S3 | A |
| H4418501 | 49613388 | 46697397 | 5493529 | 41203868 | S5 | A |
| H44581 | 17484794 | 16541829 | 2593761 | 13948068 | S1 | A |
| H44582 | 20828828 | 19724643 | 3517878 | 16206765 | S2 | A |
| H44583 | 35891558 | 34425269 | 5853110 | 28572159 | S3 | A |
| H44681 | 19533008 | 18492912 | 4510325 | 13982587 | S1 | A |
| H44682 | 22629880 | 21376405 | 4156835 | 17219570 | S2 | A |
| H44683 | 37067862 | 35705498 | 6128762 | 29576736 | S3 | A |
| H4468501 | 35122513 | 33038047 | 4159167 | 28878880 | S5 | A |
| H45781 | 20713449 | 19597581 | 3653953 | 15943628 | S1 | A |
| H45782 | 20506530 | 19449708 | 2903101 | 16546607 | S2 | A |
| H45783 | 41547215 | 39982912 | 6426876 | 33556036 | S3 | A |
| H4578501 | 34780937 | 32571327 | 4194109 | 28377218 | S5 | A |
| H46181 | 17522579 | 16670088 | 3105450 | 13564638 | S1 | A |
| H46182 | 16710665 | 15832152 | 2588890 | 13243262 | S2 | A |
| H46183 | 35705975 | 34372583 | 6103224 | 28269359 | S3 | A |
| H50581 | 19148169 | 18144977 | 3322293 | 14822684 | S1 | A |
| H5058501 | 36388480 | 34353671 | 4314215 | 30039456 | S5 | A |
| H52181 | 21723313 | 20619693 | 3364546 | 17255147 | S1 | A |
| H52182 | 35453419 | 34212816 | 5715167 | 28497649 | S2 | A |
| H52183 | 40825150 | 39301543 | 6456891 | 32844652 | S3 | A |
| H5218501 | 34670135 | 32454020 | 4632773 | 27821247 | S5 | A |
| H55481 | 20605783 | 19584462 | 2847034 | 16737428 | S1 | A |
| H55482 | 38431691 | 37078467 | 5745958 | 31332509 | S2 | A |
| H55483 | 60159993 | 58121563 | 9746136 | 48375427 | S3 | A |
| H5548501 | 33587451 | 31595405 | 3874577 | 27720828 | S5 | A |
| H58181 | 22758777 | 21608259 | 3401447 | 18206812 | S1 | A |
| H58182 | 38708233 | 37477203 | 7183280 | 30293923 | S2 | A |
| H58183 | 42112222 | 40638711 | 6520219 | 34118492 | S3 | A |
| H5818501 | 24409214 | 22840079 | 3124251 | 19715828 | S5 | A |
| H59081 | 22541587 | 21336792 | 3311067 | 18025725 | S1 | A |
| H59082 | 41653257 | 40341920 | 6172584 | 34169336 | S2 | A |
| H59083 | 47613701 | 45914566 | 6565592 | 39348974 | S3 | A |
| H5908501 | 33865792 | 31657264 | 4394258 | 27263006 | S5 | A |
| H59281 | 20379168 | 19409621 | 3392668 | 16016953 | S1 | A |
| H59282 | 31210591 | 30119950 | 5745650 | 24374300 | S2 | A |
| H59283 | 49200123 | 47457153 | 7325110 | 40132043 | S3 | A |
| H5928501 | 35447518 | 33641169 | 3738302 | 29902867 | S5 | A |
| H59481 | 21268807 | 20264491 | 3943849 | 16320642 | S1 | A |
| H59482 | 35361912 | 34143269 | 7475447 | 26667822 | S2 | A |
| H59483 | 44648130 | 43172948 | 6854368 | 36318580 | S3 | A |
| H5948501 | 36714202 | 34487196 | 4304462 | 30182734 | S5 | A |
| H59581 | 20865441 | 19860043 | 3413374 | 16446669 | S1 | A |
| H59582 | 34270152 | 33049216 | 5389990 | 27659226 | S2 | A |
| H59583 | 43129397 | 41513505 | 7588692 | 33924813 | S3 | A |
| H5958501 | 41485003 | 38886023 | 4455917 | 34430106 | S5 | A |
| H60381 | 17498279 | 16594892 | 2512306 | 14082586 | S1 | A |
| H60382 | 32557424 | 31471956 | 4633579 | 26838377 | S2 | A |
| H60383 | 53476181 | 51591321 | 7839952 | 43751369 | S3 | A |
| H6038501 | 41495545 | 38948782 | 4613320 | 34335462 | S5 | A |
| H62481 | 23484225 | 22370723 | 3745527 | 18625196 | S1 | A |
| H62482 | 40930287 | 39468019 | 5861595 | 33606424 | S2 | A |
| H62483 | 42837706 | 41297453 | 6788504 | 34508949 | S3 | A |
| H6248501 | 28207231 | 26209073 | 3429820 | 22779253 | S5 | A |
| H66781 | 19809761 | 18842030 | 2983045 | 15858985 | S1 | A |
| H66782 | 33795836 | 32597854 | 6066035 | 26531819 | S2 | A |
| H66783 | 46039945 | 44510395 | 6724797 | 37785598 | S3 | A |
| H6678501 | 33123908 | 31101269 | 4340304 | 26760965 | S5 | A |
| H70881 | 26229243 | 24984197 | 4493002 | 20491195 | S1 | A |
| H70882 | 35344200 | 34170928 | 5114906 | 29056022 | S2 | A |
| H70883 | 58456325 | 56520684 | 8735933 | 47784751 | S3 | A |
| H7088501 | 28556558 | 26512534 | 3690609 | 22821925 | S5 | A |
| H71181 | 19950029 | 18973689 | 3258056 | 15715633 | S1 | A |
| H71182 | 42352578 | 40948164 | 6691163 | 34257001 | S2 | A |
| H71183 | 45110209 | 43362143 | 9988682 | 33373461 | S3 | A |
| H7118501 | 26704526 | 24707535 | 3064646 | 21642889 | S5 | A |
| H71281 | 17356146 | 16399184 | 2656930 | 13742254 | S1 | A |
| H71282 | 38663830 | 37376340 | 5363530 | 32012810 | S2 | A |
| H71283 | 40040480 | 38543192 | 6299135 | 32244057 | S3 | A |
| H7128501 | 38522563 | 36265465 | 5005761 | 31259704 | S5 | A |
| H71381 | 17499576 | 16595859 | 2702793 | 13893066 | S1 | A |
| H71382 | 35963350 | 34698598 | 4978688 | 29719910 | S2 | A |
| H71383 | 58375547 | 55965036 | 7236633 | 48728403 | S3 | A |
| H7138501 | 33912622 | 31900522 | 4301288 | 27599234 | S5 | A |
| H71481 | 20003145 | 19005100 | 3242189 | 15762911 | S1 | A |
| H71482 | 36228208 | 34794296 | 6269317 | 28524979 | S2 | A |
| H71483 | 40285292 | 38551338 | 4954990 | 33596348 | S3 | A |
| H7148501 | 32699791 | 30788599 | 4657861 | 26130738 | S5 | A |
| H80181 | 18607483 | 17760015 | 3361055 | 14398960 | S1 | A |
| H80182 | 37170083 | 35805520 | 6736855 | 29068665 | S2 | A |
| H80183 | 49092689 | 46966913 | 6654393 | 40312520 | S3 | A |
| H8018501 | 37754595 | 35549798 | 4801380 | 30748418 | S5 | A |
| H84781 | 19613062 | 18556370 | 3702559 | 14853811 | S1 | A |
| H84782 | 44419236 | 42850183 | 6515145 | 36335038 | S2 | A |
| H84783 | 54282407 | 52232999 | 7137461 | 45095538 | S3 | A |
| H8478501 | 34210367 | 32255655 | 4959194 | 27296461 | S5 | A |
| H84881 | 18293151 | 17349808 | 3583814 | 13765994 | S1 | A |
| H84882 | 38341328 | 36954895 | 6011185 | 30943710 | S2 | A |
| H84883 | 44961229 | 43061879 | 5554313 | 37507566 | S3 | A |
| H8488501 | 32243112 | 30180040 | 4247326 | 25932714 | S5 | A |
| 211383 | 36405339 | 35207701 | 5306022 | 29901679 | S3 | B |
| 211483 | 42449411 | 40943588 | 6348934 | 34594654 | S3 | B |
| 211583 | 43449103 | 42154955 | 7079310 | 35075645 | S3 | B |
| 211683 | 40457470 | 39228576 | 6038684 | 33189892 | S3 | B |
| 211783 | 41023496 | 39820777 | 5458187 | 34362590 | S3 | B |
| 211883 | 45064882 | 43827977 | 6556386 | 37271591 | S3 | B |
| 211884 | 49779489 | 48308028 | 7054922 | 41253106 | S4 | B |
| 211983 | 49242780 | 47838096 | 7957203 | 39880893 | S3 | B |
| 211984 | 45603376 | 44389275 | 6589337 | 37799938 | S4 | B |
| 212083 | 41182608 | 39687307 | 6416188 | 33271119 | S3 | B |
| 212084 | 51108698 | 49692706 | 8240241 | 41452465 | S4 | B |
| 212183 | 52366209 | 48642317 | 6862226 | 41780091 | S3 | B |
| 212184 | 50209085 | 47874576 | 6654333 | 41220243 | S4 | B |
| 212283 | 42558499 | 40341393 | 5974602 | 34366791 | S3 | B |
| 212284 | 48006814 | 46698815 | 7632800 | 39066015 | S4 | B |
| 212383 | 50057035 | 48291599 | 7876293 | 40415306 | S3 | B |
| 212384 | 43082516 | 42014410 | 7113791 | 34900619 | S4 | B |
| 212483 | 44158202 | 42360054 | 6111350 | 36248704 | S3 | B |
| 212484 | 42644632 | 41467968 | 6811169 | 34656799 | S4 | B |
| 212583 | 41796540 | 40258441 | 6988045 | 33270396 | S3 | B |
| 212584 | 58847455 | 57048017 | 8813493 | 48234524 | S4 | B |
| 212683 | 42852435 | 41456424 | 6261178 | 35195246 | S3 | B |
| 212684 | 47402272 | 45096611 | 6814094 | 38282517 | S4 | B |
| 212783 | 48084943 | 46620119 | 7124777 | 39495342 | S3 | B |
| 212784 | 46676122 | 44330224 | 6850246 | 37479978 | S4 | B |
| 212883 | 44333654 | 42478068 | 5653019 | 36825049 | S3 | B |
| 212884 | 49469107 | 47253688 | 6132718 | 41120970 | S4 | B |
| 212984 | 52329571 | 50194040 | 7508750 | 42685290 | S4 | B |
| 213084 | 72624158 | 70063598 | 12305363 | 57758235 | S4 | B |
| 213184 | 58929710 | 57485597 | 10713312(18.64%) | -81.36% | S4 | B |
| 213284 | 48698170 | 46891993 | 7849262 | 39042731 | S4 | B |
| H42284 | 57002793 | 55592070 | 7337966 | 48254104 | S4 | A |
| H44184 | 53138701 | 51389434 | 6159415 | 45230019 | S4 | A |
| H44584 | 40972576 | 39484658 | 6406532 | 33078126 | S4 | A |
| H44684 | 54212438 | 52655946 | 8315642 | 44340304 | S4 | A |
| H45784 | 44391178 | 43400789 | 6241029 | 37159760 | S4 | A |
| H46184 | 48793775 | 47698463 | 6459520 | 41238943 | S4 | A |
| H50584 | 51760457 | 50079681 | 5536890 | 44542791 | S4 | A |
| H52184 | 49286842 | 47956169 | 6804977 | 41151192 | S4 | A |
| H55484 | 52924361 | 49354174 | 4103443 | 45250731 | S4 | A |
| H58184 | 38399959 | 37570607 | 5450383 | 32120224 | S4 | A |
| H59084 | 49421349 | 48395581 | 7933268 | 40462313 | S4 | A |
| H59284 | 48741905 | 47733460 | 8432863 | 39300597 | S4 | A |
| H59484 | 36046908 | 35355482 | 6076436 | 29279046 | S4 | A |
| H59584 | 48635769 | 47603663 | 7210837 | 40392826 | S4 | A |
| H60384 | 39789140 | 38968598 | 6026045 | 32942553 | S4 | A |
| H62484 | 38680438 | 37875270 | 4874478 | 33000792 | S4 | A |
| 210283 | 51301084 | 47247555 | 7736757 | 39510798 | S3 | B |
| 210284 | 42528093 | 39882363 | 8355515 | 31526848 | S4 | B |
| 210383 | 58656492 | 53256144 | 8156353 | 45099791 | S3 | B |
| 210384 | 27199328 | 25948106 | 4629950 | 21318156 | S4 | B |
| 210483 | 75238363 | 66060817 | 10652216 | 55408601 | S3 | B |
| 210484 | 40309954 | 38459678 | 9499464 | 28960214 | S4 | B |
| 210583 | 52742030 | 51222981 | 7195992 | 44026989 | S3 | B |
| 210584 | 79341107 | 76079820 | 16612444 | 59467376 | S4 | B |
| 210683 | 38717501 | 37163021 | 5615026 | 31547995 | S3 | B |
| 210684 | 23412011 | 22291068 | 5031202 | 17259866 | S4 | B |
| 210783 | 31777758 | 30658889 | 5351072 | 25307817 | S3 | B |
| 210784 | 18414276 | 17442318 | 3593845 | 13848473 | S4 | B |
| 210883 | 60903635 | 58640591 | 9664006 | 48976585 | S3 | B |
| 210884 | 66445149 | 62534843 | 14239265 | 48295578 | S4 | B |
| 210983 | 50885390 | 48866393 | 7206080 | 41660313 | S3 | B |
| 210984 | 43677880 | 41921692 | 9098737 | 32822955 | S4 | B |
| 211083 | 41495160 | 39870275 | 9108767 | 30761508 | S3 | B |
| 211084 | 45763332 | 43756952 | 9345168 | 34411784 | S4 | B |
| 211183 | 42490437 | 40895180 | 6149885 | 34745295 | S3 | B |
| 211184 | 44549505 | 42585938 | 11126898 | 31459040 | S4 | B |
| 211283 | 45081644 | 42933058 | 7031670 | 35901388 | S3 | B |
| 211284 | 41076120 | 39342543 | 8955330 | 30387213 | S4 | B |
| 211384 | 28010033 | 26850638 | 5954519 | 20896119 | S4 | B |
| 211484 | 86335550 | 82928020 | 17315424 | 65612596 | S4 | B |
| 211584 | 29119867 | 28009686 | 5700165 | 22309521 | S4 | B |
| 211684 | 45365289 | 43246262 | 9673791 | 33572471 | S4 | B |
| 213082 | 40318360 | 38663886 | 7228267 | 31435619 | S2 | B |
| 213182 | 46478336 | 44059403 | 7377532 | 36681871 | S2 | B |
| 213282 | 62716331 | 59008274 | 11093183 | 47915091 | S2 | B |
| 213382 | 58208100 | 52149585 | 8758374 | 43391211 | S2 | B |
| 213482 | 56439105 | 53343918 | 9617871 | 43726047 | S2 | B |
| 213485 | 15313067 | 14571028 | 2758754 | 11812274 | S5 | B |
| H66784 | 42908198 | 41274364 | 6519890 | 34754474 | S4 | A |
| H70884 | 38791571 | 37038902 | 6351377 | 30687525 | S4 | A |
| H71184 | 55562464 | 52856784 | 8709898 | 44146886 | S4 | A |
| H71284 | 37377972 | 35320585 | 7197134 | 28123451 | S4 | A |
| H71384 | 40825153 | 39340786 | 8717465 | 30623321 | S4 | A |
| H71484 | 34321752 | 32974494 | 5573907 | 27400587 | S4 | A |
| H80184 | 46843700 | 45239598 | 8846943 | 36392655 | S4 | A |
| H84784 | 33330732 | 32054216 | 5963517 | 26090699 | S4 | A |
| H84884 | 37251893 | 35705769 | 6750021 | 28955748 | S4 | A |
| 210285 | 63179655 | 61497729 | 11335153 | 50162576 | S5 | B |
| 210385 | 63198554 | 61417234 | 12113507 | 49303727 | S5 | B |
| 210485 | 53664853 | 52124164 | 8997592 | 43126572 | S5 | B |
| 210585 | 56880841 | 55136276 | 10508931 | 44627345 | S5 | B |
| 210685 | 77817136 | 75921123 | 13370022 | 62551101 | S5 | B |
| 210785 | 60758080 | 59044935 | 12519768 | 46525167 | S5 | B |
| 210885 | 58535203 | 56062784 | 12160336 | 43902448 | S5 | B |
| 210985 | 57017964 | 54501051 | 12756432 | 41744619 | S5 | B |
| 211085 | 39533379 | 37613080 | 7488976 | 30124104 | S5 | B |
| 211185 | 46761169 | 44831025 | 9057103 | 35773922 | S5 | B |
| 211285 | 60521504 | 57594609 | 10691311 | 46903298 | S5 | B |
| 211385 | 54391861 | 51737062 | 9760921 | 41976141 | S5 | B |
| 211485 | 45434769 | 43283727 | 7809447 | 35474280 | S5 | B |
| 211585 | 44894642 | 42472448 | 9048662 | 33423786 | S5 | B |
| 211685 | 50597957 | 46413016 | 9719156 | 36693860 | S5 | B |
| 211785 | 45726777 | 43097062 | 6880914 | 36216148 | S5 | B |
| 211885 | 49480789 | 46539141 | 8616604 | 37922537 | S5 | B |
| 211985 | 38061608 | 35478624 | 7222156 | 28256468 | S5 | B |
| 212085 | 60500738 | 57825003 | 10474870 | 47350133 | S5 | B |
| 212185 | 44931982 | 42389282 | 6751690 | 35637592 | S5 | B |
| 212285 | 74847740 | 70152108 | 12235201 | 57916907 | S5 | B |
| 212385 | 51562024 | 49055552 | 8692451 | 40363101 | S5 | B |
| 212485 | 48319588 | 44356702 | 7510785 | 36845917 | S5 | B |
| 212585 | 44162391 | 40999266 | 7002358 | 33996908 | S5 | B |
| 212685 | 56278886 | 51985630 | 9014073 | 42971557 | S5 | B |
| 212785 | 53142472 | 48618349 | 7153619 | 41464730 | S5 | B |
| 212885 | 50249030 | 45957627 | 7987325 | 37970302 | S5 | B |
| 212983 | 22410710 | 20749208 | 2902245 | 17846963 | S3 | B |
| 212985 | 40554174 | 36812478 | 6602827 | 30209651 | S5 | B |
| 213083 | 32841421 | 30819498 | 4602974 | 26216524 | S3 | B |
| 213085 | 38076121 | 34884135 | 5988681 | 28895454 | S5 | B |
| 213183 | 34843064 | 32930221 | 5738077 | 27192144 | S3 | B |
| 213185 | 51222123 | 47303747 | 8176669 | 39127078 | S5 | B |
| 213283 | 24655977 | 22776133 | 3265016 | 19511117 | S3 | B |
| 213285 | 20558773 | 19543610 | 3702780 | 15840830 | S5 | B |
| 213383 | 17332620 | 16266953 | 2492976 | 13773977 | S3 | B |
| 213384 | 67012962 | 65308074 | 11911638 | 53396436 | S4 | B |
| 213385 | 31314070 | 29269465 | 7112811 | 22156654 | S5 | B |
| 213483 | 23621146 | 22396812 | 3547663 | 18849149 | S3 | B |
| 213484 | 63975617 | 62236435 | 11300132 | 50936303 | S4 | B |
| H38184 | 37668007 | 35830433 | 6223963 | 29606470 | S4 | A |
| H38384 | 38041569 | 36333936 | 3945082 | 32388854 | S4 | A |
| H38884 | 49391210 | 46900782 | 5552097 | 41348685 | S4 | A |
| H40584 | 30858437 | 29589195 | 3876249 | 25712946 | S4 | A |
| H40684 | 33806258 | 32355340 | 4830059 | 27525281 | S4 | A |
| H41084 | 34638645 | 33133965 | 4821112 | 28312853 | S4 | A |
| H41184 | 24511872 | 23417311 | 3317231 | 20100080 | S4 | A |

**Supplemental Table 5.** Alpha diversity summary statistics by system and sampling point

| **Sampling Period** | **System** | **Shannon mean** | **Shannon median** | **Shannon min.** | **Shannon max.** | **Inverse Simpson mean** | **Inverse Simpson median** | **Inverse Simpson min.** | **Inverse Simpson Max** |
| --- | --- | --- | --- | --- | --- | --- | --- | --- | --- |
| 1 | A | 1.391 | 1.414 | 0.948 | 1.726 | 2.695 | 2.617 | 1.653 | 4.107 |
| 2 | A | 1.442 | 1.533 | 0.915 | 1.694 | 2.853 | 2.999 | 1.628 | 3.893 |
| 3 | A | 1.277 | 1.285 | 0.879 | 1.711 | 2.391 | 2.268 | 1.600 | 3.783 |
| 4 | A | 1.108 | 1.048 | 0.931 | 1.761 | 2.031 | 1.819 | 1.645 | 4.449 |
| 5 | A | 1.192 | 1.141 | 0.970 | 1.640 | 2.146 | 1.973 | 1.684 | 3.544 |
| 1 | B | 1.264 | 1.246 | 0.740 | 1.758 | 2.313 | 2.181 | 1.421 | 4.174 |
| 2 | B | 1.223 | 1.149 | 0.928 | 1.846 | 2.311 | 2.112 | 1.706 | 4.518 |
| 3 | B | 1.036 | 1.036 | 0.964 | 1.131 | 1.899 | 1.891 | 1.761 | 2.075 |
| 4 | B | 1.045 | 1.047 | 0.925 | 1.233 | 1.852 | 1.863 | 1.653 | 2.140 |
| 5 | B | 1.062 | 1.052 | 0.967 | 1.184 | 1.907 | 1.875 | 1.741 | 2.237 |
